# Exploring the Impact of Insertion/Deletion in *FTO* and *PLIN1* Genes on Morphometric Traits in Sheep

**DOI:** 10.3390/ani13193032

**Published:** 2023-09-27

**Authors:** Xinle Wang, Jingyun Li, Junyan Bai, Mengke Chen, Longwei Wang, Hongdeng Fan, Fanlin Zeng, Xiaoning Lu, Yuhan He

**Affiliations:** College of Animal Science and Technology, Henan University of Science and Technology, Luoyang 471023, China; wangxinle163@163.com (X.W.);

**Keywords:** InDel, *FTO*, *PLIN1*, morphometric traits, sheep

## Abstract

**Simple Summary:**

Fat mass and obesity-associated (*FTO*) and perilipin1 (*PLIN1*) genes have been associated with fat mass deposition. These genes have also been found to regulate economic traits (e.g., morphometric traits) in animals. In this study, the researchers analyzed insertion–deletion (InDel) variations in the *FTO* and *PLIN1* genes and their association with morphometric traits in three sheep breeds (Hu sheep, Dupor sheep, and Small Tail Han sheep). A total of six InDels (*FTO-2*, *FTO-3*, *FTO-4*, *FTO-5*, *FTO-6*, and *PLIN1*) were identified in the *FTO* and *PLIN1* genes in the three breeds of sheep. Genetic variations of these InDels were evaluated on the basis of their polymorphism information content (PIC). The *FTO-6* and *PLIN1* genes showed low levels of polymorphism (0 < PIC < 0.25), while the other four InDels were moderately polymorphic (0.25 < PIC < 0.50) in the three breeds of sheep. The results of the association analysis revealed that four InDels from the *FTO* and *PLIN1* genes were significantly associated with the morphometric traits in the three sheep breeds, such as body weight, body height, chest width, chest depth, cannon circumference, head length, coccyx length, forehead width, and back height. Based on these findings, the *FTO* and *PLIN1* genes can serve as genetic markers to select sheep with desirable morphometric traits.

**Abstract:**

This study aimed to identify InDels from the *FTO* and *PLIN1* genes and to analyze their association with morphometric traits in Hu sheep (HS), Dupor sheep (DS), and Small Tail Han sheep (STHS). The *FTO* and *PLIN1* genes were genotyped using the insertion/deletion (InDel) method. A one-way ANOVA with SPSS 26.0 software (IBM Corp, Armonk, NY, USA) was used to assess the effect of the InDel *FTO* and *PLIN1* genes on morphometric traits. The results revealed significant associations between certain InDels and the morphometric traits in different breeds of sheep. Specifically, *FTO-2* was significantly associated with cannon circumference (CaC) in HS rams and body height (BoH) in HS ewes (*p* < 0.05). *FTO-2* was also significantly associated with chest width (ChW), CaC, head length (HeL), and coccyx length (CoL) in the STHS breed (*p* < 0.05). *FTO-3* showed significant associations with BoH in HS rams and BoH, back height (BaH), ChW, and chest depth (ChD) in HS ewes (*p* < 0.05). *FTO-3* was also significantly associated with ChW in the DS and STHS breeds (*p* < 0.05). *FTO-5* was significantly associated with body weight (BoW) in the DS breed and BoH in the STHS breed (*p* < 0.05). Furthermore, *PLIN1* was significantly related to BoW in the DS breed and was significantly associated with CoL and forehead width (FoW) in the STHS breed (*p* < 0.05). In conclusion, the study suggested that InDels in the *FTO* and *PLIN1* genes could provide practical information to improve morphometric traits in sheep breeding.

## 1. Introduction

Hu sheep (HS), Dupor sheep (DS), and Small Tail Han sheep (STHS) are common types in the sheep farming industry in China. HS sheep have excellent reproductive performance, are adapted to factory feeding under housed conditions, and are resistant to heat and humidity. DS are precocious; they have early growth, high carcass leanness, and tender meat; and they are resistant to rough feeding and suitable for the arid and barren ecological environment. STHS are ideal for fat lamb production because of their fast growth and early sexual maturity. To better meet the needs of consumers and improve the selection of sheep, researchers need to effectively produce larger and heavier breeds of sheep with the lowest feed consumption in the shortest possible time. Marker-assisted selection has become an efficient, rapid, and reliable breeding method, which is widely used in livestock breeding research [1]. InDel is a new molecular marker that is different from SNPs; it has good stability, high polymorphism, and a simple typing system, which can improve the intensity and efficiency of selection [2,3,4,5]. By identifying the target trait to be studied, selecting multiple related genes for linkage and correlation analysis, and investigating whether the mutation is associated with the phenotype, selection accuracy is greatly improved, and sheep breeds with excellent production performance can be produced.

Among the many genes known to influence morphometric traits, the *FTO* (fat mass and obesity-associated) and *PLIN1* (perilipin 1) genes have gained attention due to their involvement in adipogenesis and lipid metabolism in the PPAR signaling pathway [6]. Both genes have been extensively studied in humans and other mammalian species, but their role in sheep remains relatively unexplored. The *FTO* gene in sheep is located on chromosome 14 and has nine exons and eight introns. Previous studies have indicated that *FTO* not only regulated obesity but was also significantly associated (*p* < 0.05) with economic traits in pigs, sheep, and cattle [7,8,9]. Wang et al. [9] reported that ten InDels of *FTO* were identified in Tong sheep; eight InDels showed a significant correlation with growth traits; and four InDels were significantly associated with fat-tailed traits (*p* < 0.05). The *PLIN1* gene in sheep is located on chromosome 18 and has nine exons and eight introns. The SNP of *PLIN* was also associated with body fat weight, low body mass index, and the total cholesterol level [10]. Furthermore, *PLIN* can also affect growth traits and carcass traits in animals. Zhang et al. [11] reported that *PLIN* had a significant effect on carcass traits in Chinese ducks and could be used as a candidate gene to select Chinese ducks for meat quality traits. Gol et al. [12] showed that *PLIN* significantly affected the lean percentage in pigs, suggesting that *PLIN* may be used as a genetic marker for lean pork growth. Zappaterra et al. [13] found that the SNP of the *PLIN* gene rs327694326 was associated with the contents of oleic acid and cis-cowpox fatty acids in the back fat tissue of large white pigs, suggesting that *PLIN* may be used as a genetic marker for the quality of pork meat. Raza et al. [14] showed that five SNPs of the *PLIN1* gene were identified in 510 Qinchuan cattle, including g.3580T > C, g.3898G > A, g.8333G > A, g.10517T > C, and g.10538G > T, and that these five SNPs were significantly associated with the depth of back fat, intramuscular fat, and chest depth in Qinchuan cattle (*p* < 0.05).

In the present study, we analyzed the polymorphisms of the *FTO* and *PLIN1* genes and their association with the morphometric traits of the HS, DS, and STHS breeds of sheep using InDel technology based on previous studies of polymorphisms for these genes in other breeds of sheep. The results may provide useful markers for the molecular breeding of sheep.

## 2. Materials and Methods

### 2.1. Ethical Treatment

All the experimental procedures in this study were approved by the Institutional Animal Care and Use Committee of the College of Animal Science, Henan University of Science and Technology (HAUST#2021-9-22), and this study was carried out in strict compliance with the guidelines of the Guide to Animal Welfare in China.

### 2.2. Experimental Animals and Morphometric Trait Data Collection

This study took three breeds of sheep (HS, DS, and STHS) as the research objects. All the individuals within the same sheep breed were healthy, unrelated, and managed under the same conditions. One hundred and ninety-two HS (110 female and 82 male), 86 DS (female), and 96 STHS (female) blood samples of three sheep breeds and dates of morphometric traits were collected at the age of six months (they were born at the same time) from native sheep meat breeding farms in Henan province. Table 1 gives sample details. Genomic DNA from 374 sheep was extracted from jugular vein blood samples by the blood genomic DNA extraction kit method (Biomed, Beijing, China) and stored at −80 °C. The morphometric traits of three sheep breeds at six months of age were measured using standard measurement methods by the same breeder in the sheep breeding farm, including body height (BoH), back height (BaH), buttock height (BuH), body length (BoL), chest circumference (ChC), chest depth (ChD), chest width (ChW), cannon circumference (CaC), waist angle width (WaW), buttock width (BuW), body weight (BoW), coccyx height (CoH), coccyx length (CoL), forelimb height (FoH), head depth (HeD), head length (HeL), neck length (NeL), abdominal circumference (AbC), forehead width (FoW), and leg hip circumference (LhC) [15].

### 2.3. Primer Designing, PCR Amplification, and Indel Genotyping

Based on the potential InDel sites of *FTO* and *PLIN1* published on the NCBI website (https://www.ncbi.nlm.nih.gov/ (accessed on 2 May 2022)), 11 specific primers were designed using Primer Premier Software (version 5.0; Premier Biosoft International, Palo Alto, CA, USA) according to the sequence of 300–500 bp before and after the InDel loci with NC_056067.1 and NC_056071.1 as reference genomes, respectively. This study aimed to identify the InDels using PCR and agarose gel electrophoresis methods. Sites with variant base numbers less than 6 bp were not detected by agarose gel electrophoresis, while sites with variant base numbers greater than 6 bp were selected. The primers were verified by the BLAST program on the NCBI website (https://blast.ncbi.nlm.nih.gov/Blast.cgi (accessed on 2 May 2022)). Primer information, product size, and InDel sizes are shown in Table 2. Three DNA pools consisting of 20 DNA samples randomly selected from three sheep breeds were constructed. The primers (Tsingke Biotechnology Co., Ltd., Beijing, China) were diluted to 10 ng/µL according to the instructions.

Polymerase chain reaction (PCR) was performed in a 10 μL reaction volume consisting of 1.0 μL of DNA, 5 μL of 2 × Taq PCR Master Mix (Dingguo Changsheng Biotechnology Co., Ltd., Beijing, China), 0.8 μL of each primer, and 2.4 μL of double-distilled water (ddH_2_O) to make up the volume. The PCR amplification procedure was as follows: pre-denaturation at 95 °C for 5 min, followed by 10 cycles of denaturation at 95 °C for 40 s; annealing at 60 °C for 30 s (started at 60 °C and decreased by 1 °C per cycle); extension at 72 °C for 45 s; 25 cycles of denaturation at 95 °C for 40 s; annealing temperature for 30 s, extension at 72 °C for 45 s; and final extension at 72 °C for 5 min. All available individuals were genotyped using 3.0% agarose gel electrophoresis. Genotyping was performed by observing the bands in which one band was the II (insertion/insertion) type or DD (deletion/deletion) and the ID (insertion/deletion) type. The amplified samples were sent to Tsingke Biotechnology Co., Ltd. (Beijing, China) for sequencing. Sequence alignment and InDel identification were performed using the MegAlign program (version 5.0; DNAstar, Madison, WI, USA). Chromas software (version 2.2.2; Technelysium, Queensland, Australia) was used to perform the sequence analyses.

### 2.4. Population Genetic Analyses

All individual genotypes at each InDel were statistically analyzed using Microsoft Excel (Microsoft Office 365, Redmond, WA, USA). The population genetic parameters of the InDel variants, such as gene frequencies, allele frequencies, homozygosity (Ho), heterozygosity (He), effective allele numbers (Ne), and polymorphism information content (PIC), were calculated by POPGENE (version 1.32) [16]. The existence of the Hardy–Weinberg equilibrium (HWE) for InDels was tested using the online SHEsis website (http://analysis.bio-x.cn/myAnalysis.php (accessed on 10 January 2023)) [17]. Linkage disequilibrium (LD) and haplotype analysis were performed using the SHEsis online website [18].

### 2.5. Statistical Analysis

All the data were in a normal distribution, which indicated suitability for subsequent analysis. Associations between the *FTO* and *PLIN1* InDel genotypes and morphometric traits were evaluated with a one-way analysis of variance (=three genotypes and sample size greater than 3) and independent sample *t*-test (=two genotypes) in SPSS Statistics (Version 26.0; IBM Corp, Armonk, NY, USA). When the analysis of variance performed on each group of genotypes indicated a significant (*p* < 0.05) difference, statistical differences between the two genotypes were subsequently evaluated with the Bonferroni correction test. All results were expressed as mean ± standard error (SE). The model was used as follows:

Y_ij_ = μ + G_i_ + e_ij_. (1)

Y_ij_: phenotypic values of morphometric traits; µ: the overall population mean of each trait; G_i_: the effect of the genotype; e_ij_: the random residual error. The age, sampling season, and rearing environment were consistent, and their effects were not considered in this model. Differences were considered significant at *p* ≤ 0.05.

## 3. Results 

### 3.1. Identification of InDel Variants in the FTO and PLIN1 Genes

**Figure 1:**
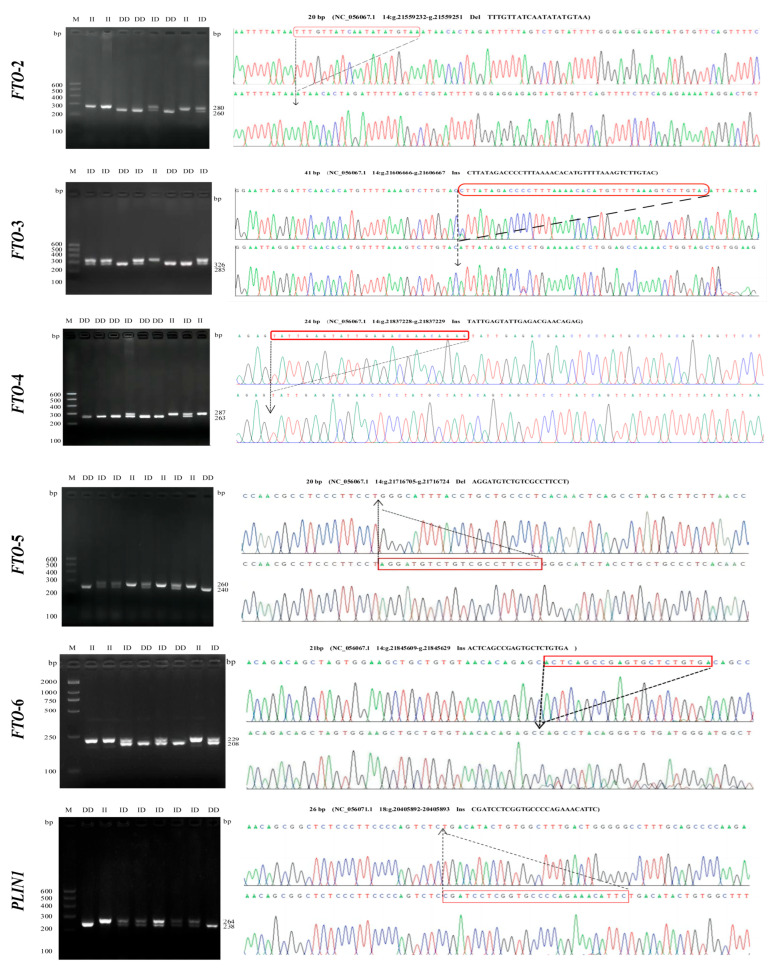
Electrophoresis pattern and sequence maps of six InDels in the *FTO* and *PLIN1* genes.

**Figure 2:**
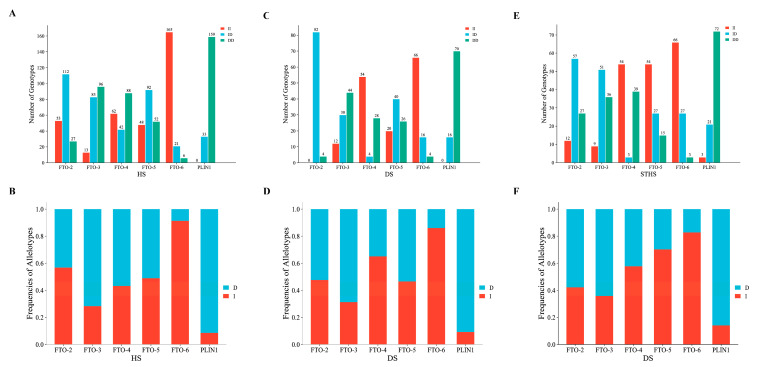
The number of genotypes and frequencies of allelotypes in the *FTO* and *PLIN1* genes. (**A**) The number of genotypes in the *FTO* and *PLIN1* genes of Hu sheep. (**B**) Frequency of allelotypes in the *FTO* and *PLIN1* genes of Hu sheep. (**C**) The number of genotypes in the *FTO* and *PLIN1* genes of Dupor sheep. (**D**) Frequency of allelotypes in the *FTO* and *PLIN1* genes of Dupor sheep. (**E**) The number of genotypes in the *FTO* and *PLIN1* genes of Small Tail Han sheep. (**F**) Frequency of allelotypes in the *FTO* and *PLIN1* genes of Small Tail Han sheep.

After the PCR amplification and sequencing of 11 potential InDel loci, a total of six InDels of the *FTO* and *PLIN1* genes (*FTO*-*2*, *FTO*-*3*, *FTO*-*4*, *FTO-5*, *FTO*-*6*, and *PLIN1*) were identified in three breeds of sheep. Each InDel locus was present in two or three genotypes: the insertion/insertion genotype (II), insertion/deletion (ID) genotype, and deletion/deletion (DD) genotype (Figure 1). Sequencing revealed a 20 bp deletion (TTTGTTATCAATATATATGTAA) in the *FTO*-*2* gene, located at NC_056067.1 g.21559232-g.21559251. A 41 bp insertion (CTTATAGACCCCTTTAAAACACATGTTTTAAAGTCTTGTAC) was identified in the *FTO-3* gene, located at NC_056067.1 g.21606666-g.21606667. A 24 bp insertion (TATTGAGTATTGAGACGAACAGAG) was identified in the *FTO-4* gene, located at NC_056067.1 g.21837228-g.21837229. A 20 bp deletion (AGGATGTCTGTCGCCTTCCT) was detected in the *FTO*-*5* gene, located at NC_056067.1 g.21716705-g.21716724. A 21 bp insertion (ACTCAGCCGAGTGCTCTGTGA) was identified in the *FTO*-6 gene, located at NC_056067.1 g.21845609-g.21845629. Furthermore, a 26 bp segment was missing in the *PLIN1* gene, which was located at NC_056071.1 g.20405892-g.20405893, and the missing sequence was CGATCCTCGGTGCCCCAGAAACATTC (Figure 1). These results were identical to those predicted for the InDel locus (on chromosome 14) of the *FTO* gene (NC_056067.1) and the InDel locus (on chromosome 18) of the *PLIN1* gene (NC_056071.1) in the NCBI database for sheep. Three genotypes (II, ID, and DD) were identified in the *FTO*-*2*, *FTO*-*3*, *FTO*-*4*, *FTO*-*5*, and *FTO*-*6* genes in the HS, DS, and STHS breeds, except that only the ID and DD genotypes were identified in the *FTO-2* gene in the DS breed. Additionally, three genotypes were identified in the *PLIN1* gene in the STHS breed, and the ID and DD genotypes were identified in the *PLIN1* gene in the HS and DS breeds (Figure 2).

### 3.2. Estimation of Genetic Parameters for the InDels of the FTO and PLIN1 Genes

The population genetic parameters were estimated for the *FTO* and *PLIN1* genes (Table 3). In the HS population, the I allele was the dominant allele in *FTO-2* and *FTO-6*, and the D allele was the dominant allele in *FTO-3*, *FTO-4*, *FTO-5*, and *PLIN1*. In the DS population, the I allele was the dominant allele in the *FTO-4* and *FTO-6*, and the D allele was the dominant allele in *FTO-2*, *FTO-3*, *FTO-5*, and *PLIN1*. In the STHS population, the I allele was the dominant allele in *FTO-4*, *FTO-5*, and *FTO-6*, and the D allele was the dominant allele in *FTO-2*, *FTO-3*, and *PLIN1* (Figure 2). Based on PIC, *FTO-6* and *PLIN1* had low polymorphism (0 < PIC < 0.25). The other three InDels were moderately polymorphic (0.25 < PIC < 0.50). A related study of the Tong sheep breed showed results similar to those of the present study. All 10 InDels detected were moderately polymorphic (0.25 < PIC < 0.50), which may be related to environmental factors and genetic differences between the breeds. Except for *FTO-2* in the three populations, *FTO-4* in the HS population, *FTO-5* in the STHS population, *FTO-6* in the HS and DS populations, which deviated from HWE (*p* < 0.05), the remaining groups were in HWE (*p* > 0.05). The homozygosity of the six InDels (*FTO-2*, *FTO-3*, *FTO-4*, *FTO-5*, *FTO-6,* and *PLIN1*) was higher than the heterozygosity, indicating a small degree of genetic variation. The maximum number of effective alleles in *FTO-5* was 1.999 in the HS breed, which suggested that the genetic drift was minimal, and the alleles were evenly distributed in the population.

### 3.3. Linkage Disequilibrium Analysis for InDels in the FTO Gene

**Figure 3:**
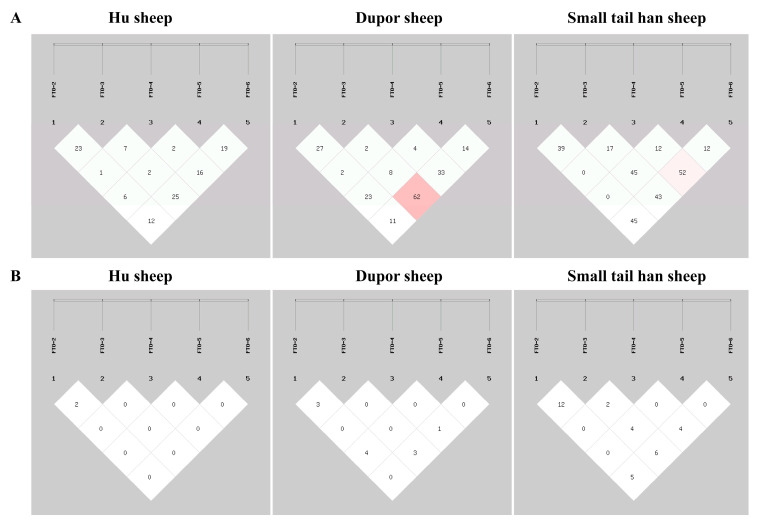
Linkage analysis of five InDels of *FTO* in three populations. (**A**) D’ analysis of three sheep breeds in five *FTO* InDels; (**B**) r^2^ analysis of three sheep breeds in five *FTO* InDels.

The linkage disequilibrium analysis of the five *FTO* InDels using the SHEsis online website showed that the five InDels are not strongly linked in the HS, DS, and STHS breeds (D^′^ > 0.88 or r^2^ > 0.33 indicates strong linkage disequilibrium), as shown in Figure 3. It indicated that these loci tend to be inherited independently [19]; so, only a single InDel locus was used for association with the growth traits of the three sheep breeds without haplotype analysis.

### 3.4. Association of InDels in the FTO and PLIN1 Genes with Morphometric Traits

The results of the association analyses of the *FTO* and *PLIN1* polymorphisms and morphometric traits in the three sheep breeds are shown in Table 4. *FTO-2* was significantly associated with CaC in HS rams (*p* < 0.05). Individuals with the ID genotype had higher CaC. *FTO-2* was significantly associated with BoH in HS ewes (*p* < 0.05). Individuals with the DD genotype had higher BoH. *FTO-2* was significantly associated with ChW, CaC, HeL, and CoL in the STHS breed (*p* < 0.05). Individuals with the II genotype had higher ChW, CaC, HeL, and CoL. *FTO-3* showed a significant association with BoH in HS rams (*p* < 0.05). Individuals with the ID genotype had higher BoH. *FTO-3* was also significantly associated with BoH, BaH, ChW, and ChD in HS ewes (*p* < 0.05). Individuals with the II genotype had higher BoH, BaH, ChW, and ChD. *FTO-3* showed a significant association with ChW in the DS and STHS breeds (*p* < 0.05). Individuals with the ID genotype had higher ChW in the DS breed, while individuals with the DD genotype had higher ChW in the STHS breed. *FTO-5* was significantly associated with BoW in the DS breed (*p* < 0.05). Individuals with the II genotype had higher BoW. *FTO-5* was also significantly associated with BoH in the STHS breed (*p* < 0.05). Individuals with the ID genotype had higher BoH. *PLIN1* was significantly related to BoW in the DS breed (*p* < 0.05). Individuals with the ID genotype had higher BoW. *PLIN1* was also significantly associated with CoL and FoW in the STHS breed (*p* < 0.05). Individuals with the ID genotype had higher CoL and FoW. No significant differences were observed between the rest of the InDels for the other morphometric traits (*p* > 0.05, Tables S1–S3). The *FTO* and *PLIN1* genes are expected to be genetic selection markers for morphometric traits in sheep.

## 4. Discussion

The economic value of livestock is heavily influenced by their growth and slaughter characteristics, and the early selection of individuals with superior phenotypes based on their genotype can greatly enhance population efficiency. In this study, the association between *FTO* and *PLIN1* polymorphisms and morphometric traits in 374 sheep individuals was examined. Six InDels in the *FTO* and *PLIN1* genes were identified and found to be correlated with morphometric traits in Hu, Small Tail Han, and Dupor sheep. This discovery offers a potential molecular marker for enhancing sheep breeding.

*FTO* is the first gene linked to human obesity; it regulates energy metabolism and food intake, leading to obesity in animals [20,21,22]. *PLIN1* is a phosphorylatable phosphoprotein associated with lipid droplets; it is involved in lipid catabolism, which is also linked to obesity [23]. Previous studies have shown that *FTO* and *PLIN1* do not only play a role in regulating obesity; they are also significantly associated (*p* < 0.05) with economically important traits in pigs, sheep, and cattle [7,8,9]. Wu et al. [24] conducted InDel detection and association analysis in seven Chinese sheep breeds, revealing a significant association between *GHR*-InDel and growth traits (*p* < 0.05). Yang et al. [25] found significant associations between seven SNPs (g.102G > A, g.255G > A, g.349C > T, g.384A > G, g.386G > A, g.444G > A, g.556G > A) of *G0S2* and carcass traits in chickens. Specifically, individuals with the AG genotype at g.444G > A exhibited the highest breast muscle weight, while individuals with the AA genotype at g.556G > A showed the lowest fat weight (*p* < 0.05). Sun et al. [26] discovered significant associations between the SNPs of *AGPAT3* and various milk production traits in Chinese Holstein cows, including test-day milk yield, protein percentage, fat percentage, 305-day milk yield, milk urea nitrogen, and somatic cell score. Huang et al. [27] demonstrated a significant association between a novel InDel of *Cry2* and litter size in Australian White sheep. Specifically, individuals with the II genotype had larger litter sizes than those with the ID genotype in P2-Del-6-bp at first parity, while individuals with the ID genotype had larger litter sizes than those with the II genotype in P1-Del-6-bp at third parity (*p* < 0.05). Liu et al. [28] identified 15 SNPs in the *GDF9* gene in Dongxiang blue-shelled chickens and Luhua chickens. Among these, three SNPs (g.17156387C > T, g.17156427A > G, and g.17156703A > C) exhibited significant associations with age at the first egg, the weight of the first egg, egg weight at 300 days of age, and the total number of eggs at 300 days of age in chickens. These studies provide evidence that mutations can induce alterations in domestic animal economic traits.

Identifying the causal variant of a gene can enhance the accuracy of genomic selection and is considered to be an efficient approach for analyzing the associations between genetic polymorphisms and economically important traits [29]. This study primarily focused on the association between mutations at the InDel locus and the morphometric traits in three sheep breeds; the traits varied within the breeds. These results might result from the differences in the genetic backgrounds of the different breeds [30]. Wang et al. [9] observed three genotypes (II, ID, and DD) at ten novel InDels of *FTO* in Tong sheep, which were similar to those of this study. These results indicate that the frequency of InDels varies across different species and is associated with differentiation, gene penetration, genomic segment size, and effective population size. In our study, there was a significant association (*p* < 0.05) between the four InDels (*FTO-2*, *FTO-3*, *FTO-5*, *PLIN1*) we detected in *FTO* and *PLIN1*, with morphometric traits in the HS, DS, and STHS breeds. This result was similar to those of previous findings in other species, where mutations in these genes were significantly associated with the animals’ growth traits [7,28,29]. The sheep industry in China has made significant progress in recent years and has primarily focused on fulfilling the daily demands of consumers. However, there is a need for additional valuable molecular genetic markers to enhance sheep growth and meat quality traits in China.

## 5. Conclusions

In summary, we identified six InDels from three sheep populations, including five InDels in *FTO* and one InDel in *PLIN1*. Three InDels in *FTO* were associated with HS, DS, and STHS growth traits, and an InDel in *PLIN1* was associated with DS and STHS morphometric traits. Our findings implied that the InDels of the *FTO* and *PLIN1* genes could be used as molecular markers for sheep breeding.

## Figures and Tables

**Table 1:** Sampling source information of three sheep breeds in this study.

Breed	Abbreviation	Sampling Source	Number	Sample Tissue
Hu	HS	Luoyang city	192	Venous blood
Dupor	DS	Luoyang city	86	Venous blood
Small Tail Han	STHS	Puyang city	96	Venous blood

**Table 2:** Primer information, product size, and InDel sizes of the sheep *FTO* and *PLIN1* genes. F for forward primer, R for reverse primer.

Gene	Primer Sequence (5′-3′)	Product Size (bp)	InDel Sizes (bp)
*FTO-1*	F: TAAGTTTACTAAGCCGAGGAGC R: TTTAGTCTGTATTTTGGGAGGA	260	37
*FTO-2*	F: TTATTGGAGTTTATTGGAGTTAC R: GTCTGTGAGCCTATTTCTGTC	280	20
*FTO-3*	F: CCACAACTCAGCAGCTACCAGT R: CTCAGCAATCATCAGCATCAGG	326	41
*FTO-4*	F: AAGGAGGTAAAGGAGAAAACT R: TACAGCAAAATGAATCTGACA	287	24
*FTO-5*	F: CCAAGGAGAAAGCTGCTGTAGTC R: GGATTTCAAAGAGATTTCACCGTTG	260	20
*FTO-6*	F: CCATCCCATCCCACCCTGTA R: GCTGCCTTGCGGACCTTT	208	21
*FTO-7*	F: CTGCATGTGCCTCGAGTCTT R: GGTCAGAGAGTTGGTGTCATGG	225	24
*FTO-8*	F: TCTTCACGGTTCATGGTAGGC R: CACTAGTGGGAATGAAGGGGAC	316	48
*FTO-9*	F: TGTGGAATTCATTCTTGTCGCT R: AGGGAGAGATGCTAAGGTCCA	304	15
*FTO-10*	F: TGGGACAGTACAGTCTTGTGTT R: TCTGGATCAACCTCCACTGTT	342	41
*PLIN1*	F: GCAGCAGGTAGGAAGGATGG R: TCACTGACAACGTGGTGGAC	238	26

**Table 3:** Allele frequencies and estimates of population parameters for InDels in the *FTO* and *PLIN1* genes.

Breed	Name	Loci	Allele Frequency		Genetic Parameter				HWE (P)
			I	D	Ho	He	PIC	Ne	
HS	*FTO-2* (P2-Del-20 bp)	NC_056067.1 Chr:14 g.21559232-g.21559251	0.568	0.432	0.509	0.491	0.370	1.964	0.009
	*FTO-3* (P3-Ins-41 bp)	NC_056067.1 Chr:14 g.21606666-g.21606667	0.284	0.716	0.593	0.407	0.324	1.685	0.381
	*FTO-4* (P4-Ins-24 bp)	NC_056067.1 Chr:14 g.21837228-g.21837229	0.432	0.568	0.509	0.491	0.370	1.964	0.000
	*FTO-5* (P5-Del-20 bp)	NC_056067.1 Chr:14 g.21716705-g.21716724	0.490	0.510	0.500	0.500	0.375	1.999	0.568
	*FTO-6* (P6-Ins-21 bp)	NC_056067.1 Chr:14 g.21845609-g.21845629	0.914	0.086	0.843	0.157	0.145	1.186	0.000
	*PLIN1* (P1-Ins-26 bp)	NC_056071.1 Chr:14 g.20405892-g.20405893	0.086	0.914	0.843	0.157	0.145	1.186	0.193
DS	*FTO-2* (P2-Del-20 bp)	NC_056067.1 Chr:14 g.21559232-g.21559251	0.477	0.523	0.501	0.499	0.374	1.996	0.000
	*FTO-3* (P3-Ins-41 bp)	NC_056067.1 Chr:14 g.21606666-g.21606667	0.314	0.686	0.569	0.431	0.338	1.757	0.078
	*FTO-4* (P4-Ins-24 bp)	NC_056067.1 Chr:14 g.21837228-g.21837229	0.651	0.349	0.546	0.454	0.351	1.833	1.000
	*FTO-5* (P5-Del-20 bp)	NC_056067.1 Chr:14 g.21716705-g.21716724	0.465	0.535	0.502	0.498	0.374	1.990	0.545
	*FTO-6* (P6-Ins-21 bp)	NC_056067.1 Chr:14 g.21845609-g.21845629	0.860	0.140	0.760	0.240	0.211	1.316	0.037
	*PLIN1* (P1-Ins-26 bp)	NC_056071.1 Chr:14 g.20405892-g.20405893	0.093	0.907	0.831	0.169	0.155	1.203	0.342
STHS	*FTO-2* (P2-Del-20 bp)	NC_056067.1 Chr:14 g.21559232-g.21559251	0.422	0.578	0.512	0.488	0.369	1.952	0.033
	*FTO-3* (P3-Ins-41 bp)	NC_056067.1 Chr:14 g.21606666-g.21606667	0.359	0.641	0.540	0.460	0.354	1.853	0.132
	*FTO-4* (P4-Ins-24 bp)	NC_056067.1 Chr:14 g.21837228-g.21837229	0.578	0.422	0.512	0.488	0.369	1.952	1.000
	*FTO-5* (P5-Del-20 bp)	NC_056067.1 Chr:14 g.21716705-g.21716724	0.703	0.297	0.583	0.417	0.330	1.717	0.001
	*FTO-6* (P6-Ins-21 bp)	NC_056067.1 Chr:14 g.21845609-g.21845629	0.828	0.172	0.715	0.285	0.244	1.398	0.906
	*PLIN1* (P1-Ins-26 bp)	NC_056071.1 Chr:14 g.20405892-g.20405893	0.141	0.859	0.758	0.242	0.212	1.319	0.352

Notes: STHS, Small Tail Han sheep; HS, Hu sheep; DS, Dupor; I, insertion; D, deletion; Ho, homozygosity; He, heterozygosity; PIC, polymorphism information content; Ne, effective allele numbers; HWE, Hardy–Weinberg equilibrium.

**Table 4:** Association of InDels in the *FTO* and *PLIN1* genes with growth traits.

Loci	Breeds	Growth Traits (cm)	Observed Genotypes (Mean ± SE)			ANOVA *p* Values	Unadjusted *p* Values	Adjusted *p* Values
			II	ID	DD			
*FTO-2*	HS ram	Cannon circumference	7.005 ± 0.168 ^ab^	7.331 ± 0.112 ^a^	6.480 ± 0.237 ^b^	*p* = 0.007	*p* = 0.008	*p* = 0.003
	HS ewe	Body height	67.359 ± 0.814 ^b^	68.369 ± 0.607 ^ab^	71.059 ± 1.362 ^a^	*p* = 0.041	*p* = 0.037	*p* = 0.012
	STHS	Chest width	33.750 ± 1.548 ^a^	26.895 ± 1.095 ^b^	27.333 ± 1.481 ^ab^	*p* = 0.033	*p* = 0.031	*p* = 0.010
	STHS	Cannon circumference	11.500 ± 0.646 ^a^	9.579 ± 0.289 ^b^	9.222 ± 0.465 ^b^	*p* = 0.020	*p* = 0.021	*p* = 0.007
	STHS	Head length	23.750 ± 1.250 ^a^	19.368 ± 0.693 ^b^	20.778 ± 0.722 ^ab^	*p* = 0.022	*p* = 0.022	*p* = 0.007
	STHS	Coccyx length	28.500 ± 2.102 ^a^	22.632 ± 0.714 ^b^	22.222 ± 1.064 ^b^	*p* = 0.007	*p* = 0.008	*p* = 0.003
*FTO-3*	HS ram	Body height	54.714 ± 1.149 ^b^	58.647 ± 0.529 ^a^	58.049 ± 0.571 ^ab^	*p* = 0.024	*p* = 0.019	*p* = 0.006
	HS ewe	Body height	72.750 ± 2.105 ^a^	69.663 ± 0.613 ^a^	66.982 ± 0.673 ^b^	*p* = 0.002	*p* = 0.013	*p* = 0.004
	HS ewe	Back height	72.167 ± 1.493 ^a^	69.643 ± 0.625 ^a^	67.364 ± 0.656 ^b^	*p* = 0.008	*p* = 0.039	*p* = 0.013
	HS ewe	Chest width	26.333 ± 3.451 ^a^	22.000 ± 0.747 ^ab^	20.200 ± 0.583 ^b^	*p* = 0.010	*p* = 0.016	*p* = 0.005
	HS ewe	Chest depth	38.333 ± 1.542 ^a^	35.490 ± 0.548 ^ab^	33.836 ± 0.510 ^b^	*p* = 0.007	*p* = 0.021	*p* = 0.007
	DS	Chest width	20.700 ± 2.382 ^b^	26.433 ± 0.543 ^a^	24.659 ± 0.751 ^ab^	*p* = 0.006	*p* = 0.005	*p* = 0.002
	STHS	Chest width	29.667 ± 2.963 ^ab^	25.765 ± 1.006 ^b^	30.417 ± 1.417 ^a^	*p* = 0.030	*p* = 0.032	*p* = 0.011
*FTO-5*	DS	Body weight	57.780 ± 1.053 ^a^	52.935 ± 1.042 ^b^	53.873 ± 1.598 ^ab^	*p* = 0.039	*p* = 0.037	*p* = 0.012
	STHS	Body height	64.000 ± 2.457 ^b^	74.056 ± 2.337 ^a^	69.200 ± 4.042 ^ab^	*p* = 0.043	*p* = 0.042	*p* = 0.014
*PLIN1*	DS	Body weight	—	57.631 ± 1.013 ^a^	53.594 ± 0.874 ^b^	*p* = 0.040	—	—
	STHS	Coccyx length	—	26.143 ± 1.682 ^a^	22.583 ± 0.645 ^b^	*p* = 0.039	—	—
	STHS	Forehead width	—	15.000 ± 0.787 ^a^	12.521 ± 0.382 ^b^	*p* = 0.008	—	—

Notes: HS ram, Hu sheep ram; HS ewe, Hu sheep ewe; STHS, Small Tail Han sheep; DS, Dupor; SE, standard error; II, insertion/insertion; ID, insertion/deletion; DD, deletion/deletion. ^a,b^ Mean values with unlike letters were significantly different, *p* < 0.05. Bonferroni multiple comparisons were performed only if there was a difference in variance analysis.

## Data Availability

All data from this study are included in Supplementary Materials.

## Supplementary Materials

The following supporting information can be downloaded at: https://www.mdpi.com/article/10.3390/ani13193032/s1. Table S1: Relationship between InDels of *FTO* and *PLIN1* and morphometric traits in HS rams. Table S2: Relationship between InDels of *FTO* and *PLIN1* and morphometric traits in HS ewes. Table S3: Relationship between InDels of *FTO* and *PLIN1* and morphometric traits in DS. Table S4: Relationship between InDels of *FTO* and *PLIN1* and morphometric traits in STHS.
