# Supplementary material for: Exploring the Impact of Insertion/Deletion in FTO and PLIN1 Genes on Morphometric Traits in Sheep
Source: Animals (Basel). 2023 Sep 27;13(19):3032. doi: 10.3390/ani13193032 (PMC10571888; doi:10.3390/ani13193032)
Supplement: Supplementary file 1 [file animals-13-03032-s001.zip › animals-2569394-supplementary.pdf]

Table S1. RelationshiP between InDels of *FTO* and *PLIN1* and morphometric traits in HS rams.

Loci	Morphometric Traits	Observed GenotyPes (Mean ± SE)				*UnadjustedP* values	*Adjusted* *P* values
		II	*ID*	*DD*	*P* values		
FTO-2	Body height (cm)	57.762±0.654	58.451±0.494	56.300±1.291	*P*=0.193	-	-
	Back height (cm)	58.714±0.769	58.667±0.502	56.400±1.035	*P*=0.168	-	-
	Buttock height (cm)	59.190±0.930	59.333±0.507	56.500±0.671	*P*=0.083	-	-
	Body length (cm)	64.524±1.039	65.725±0.788	62.700±1.325	*P*=0.226	-	-
	Chest circumference (cm)	68.952±0.844	69.598±0.733	68.500±1.232	*P*=0.745	-	-
	Chest width (cm)	16.905±0.351	17.275±0.286	15.800±0.442	*P*=0.079	-	-
	Chest depth (cm)	28.524±0.620	28.784±0.375	27.600±0.340	*P*=0.419	-	-
	Cannon circumference (cm)	7.005±0.168[ab]	7.331±0.112[a]	6.480±0.237[b]	*P*=0.007	*P*=0.008	*P*=0.003
FTO-3	Body height (cm)	54.714±1.149[b]	58.647±0.529[a]	58.049±0.571[ab]	*P*=0.024	*P*=0.019	*P*=0.006
	Back height (cm)	55.571±1.556	59.029±0.526	58.366±0.580	*P*=0.064	-	-
	Buttock height (cm)	57.286±1.267	59.088±0.631	59.122±0.601	*P*=0.472	-	-
	Body length (cm)	64.429±2.010	63.647±0.691	66.317±0.937	*P*=0.089	-	-
	Chest circumference (cm)	66.714±1.267	69.044±0.755	69.951±0.798	*P*=0.230	-	-
	Chest width (cm)	16.857±0.261	16.941±0.330	17.073±0.323	*P*=0.938	-	-
	Chest depth (cm)	27.714±0.680	28.147±0.439	29.073±0.417	*P*=0.201	-	-
	Cannon circumference (cm)	6.857±0.322	7.144±0.164	7.193±0.112	*P*=0.619	-	-
FTO-4	Body height (cm)	57.158±0.828	58.833±0.636	57.923±0.584	*P*=0.293	-	-
	Back height (cm)	57.737±0.949	59.958±0.627	57.769±0.537	*P*=0.038	-	-
	Buttock height (cm)	58.053±0.877	59.917±0.771	58.795±0.580	*P*=0.252	-	-
	Body length (cm)	64.105±1.587	66.917±0.905	64.359±0.756	*P*=0.120	-	-
	Chest circumference (cm)	67.684±1.254	70.854±0.783	69.128±0.749	*P*=0.088	-	-
	Chest width (cm)	17.000±0.607	17.167±0.389	16.897±0.240	*P*=0.866	-	-
	Chest depth (cm)	28.842±0.526	28.708±0.540	28.359±0.437	*P*=0.768	-	-
	Cannon circumference (cm)	7.053±0.157	7.179±0.166	7.167±0.147	*P*=0.862	-	-
FTO-5	Body height (cm)	57.824±0.796	57.848±0.543	58.579±0.770	*P*=0.728	-	-
	Back height (cm)	58.235±0.834	58.326±0.532	58.737±0.865	*P*=0.896	-	-
	Buttock height (cm)	59.000±0.974	58.587±0.556	59.789±0.786	*P*=0.502	-	-
	Body length (cm)	63.647±1.303	64.891±0.661	66.684±1.556	*P*=0.221	-	-
	Chest circumference (cm)	68.529±1.105	69.467±0.643	69.579±1.343	*P*=0.755	-	-
	Chest width (cm)	16.588±0.272	16.957±0.247	17.474±0.646	*P*=0.376	-	-
	Chest depth (cm)	27.706±0.427	28.978±0.415	28.368±0.578	*P*=0.208	-	-
	Cannon circumference (cm)	7.000±0.171	7.109±0.114	7.358±0.238	*P*=0.399	-	-
FTO-6	Body height (cm)	58.151±0.409	56.889±1.184	-	*P*=0.311	-	-
	Back height (cm)	58.589±0.415	56.889±1.218	-	*P*=0.180	-	-
	Buttock height (cm)	59.164±0.436	57.222±1.164	-	*P*=0.142	-	-
	Body length (cm)	65.096±0.620	64.667±1.900	-	*P*=0.820	-	-
	Chest circumference (cm)	69.322±0.548	69.111±1.814	-	*P*=0.901	-	-
	Chest width (cm)	17.055±0.223	16.556±0.669	-	*P*=0.463	-	-
	Chest depth (cm)	28.685±0.308	27.667±0.687	-	*P*=0.268	-	-
	Cannon circumference (cm)	7.121±0.100	7.333±0.210	-	*P*=0.471	-	-
PLIN1	Body height (cm)	-	58.333±1.022	57.940±0.418	*P*=0.697	-	-
	Back height (cm)	-	59.533±1.077	58.149±0.416	*P*=0.177	-	-
	Buttock height (cm)	-	58.667±0.903	59.015±0.465	*P*=0.746	-	-
	Body length (cm)	-	64.667±1.767	65.134±0.606	*P*=0.760	-	-
	Chest circumference (cm)	-	69.433±1.556	69.269±0.544	*P*=0.904	-	-
	Chest width (cm)	-	17.200±0.835	16.955±0.183	*P*=0.657	-	-
	Chest depth (cm)	-	29.000±0.683	28.478±0.316	*P*=0.483	-	-
	Cannon circumference (cm)	-	7.300±0.315	7.109±0.088	*P*=0.423	-	-

Notes: HS, Hu sheeP; SE, standard error; II, insertion/insertion; ID, insertion/deletion; DD, deletion/deletion. [a,b]Mean values with unlike letters were significantly different, $P < 0.05$. Bonferroni multiple comparisons were performed only if there was a difference in variance analysis.

Table S2. RelationshiP between InDels of *FTO* and *PLIN1* and morphometric traits in HS ewes.

Loci	Morphometric Traits	Observed GenotyPes (Mean ± SE)				*Unadjusted*	*Adjusted*
		II	*ID*	*DD*	*P* values	*P* values	*P* values
FTO-2	Body height (cm)	67.359±0.814[b]	68.369±0.607[ab]	71.059±1.362[a]	*P*=0.041	*P*=0.037	*P*=0.012
	Back height (cm)	67.813±0.744	68.615±0.613	70.294±1.331	*P*=0.223	-	-
	Buttock height (cm)	67.969±0.790	68.926±0.605	71.441±1.320	*P*=0.055	-	-
	Body length (cm)	78.969±1.281	80.639±1.039	83.412±2.072	*P*=0.180	-	-
	Chest circumference (cm)	83.972±1.690	87.641±1.428	90.000±3.266	*P*=0.153	-	-
	Chest width (cm)	20.313±0.659	21.393±0.686	23.059±1.592	*P*=0.210	-	-
	Chest depth (cm)	33.781±0.497	35.131±0.558	35.647±0.981	*P*=0.189	-	-
	Cannon circumference (cm)	8.084±0.153	8.297±0.125	8.365±0.222	*P*=0.499	-	-
FTO-3	Body height (cm)	72.750±2.105[a]	69.663±0.613[a]	66.982±0.673[b]	*P*=0.002	*P*=0.013	*P*=0.004
	Back height (cm)	72.167±1.493[a]	69.643±0.625[a]	67.364±0.656[b]	*P*=0.008	*P*=0.013	*P*=0.004
	Buttock height (cm)	72.333±1.647	69.755±0.709	68.036±0.624	*P*=0.044	-	-
	Body length (cm)	85.667±2.124	81.663±1.209	79.064±1.015	*P*=0.069	-	-
	Chest circumference (cm)	92.333±2.305	87.851±1.599	85.536±1.554	*P*=0.278	-	-
	Chest width (cm)	26.333±3.451[a]	22.000±0.747[ab]	20.200±0.583[b]	*P*=0.010	*P*=0.016	*P*=0.005
	Chest depth (cm)	38.333±1.542[a]	35.490±0.548[ab]	33.836±0.510[b]	*P*=0.007	*P*=0.021	*P*=0.007
	Cannon circumference (cm)	8.450±0.138	8.457±0.121	8.035±0.137	*P*=0.060	-	-
FTO-4	Body height (cm)	67.779±0.644	70.778±1.228	68.276±0.754	*P*=0.088	-	-
	Back height (cm)	68.407±0.651	69.778±1.095	68.429±0.747	*P*=0.546	-	-
	Buttock height (cm)	68.895±0.683	69.417±1.109	69.020±0.756	*P*=0.930	-	-
	Body length (cm)	80.826±1.205	81.444±1.946	80.051±1.159	*P*=0.795	-	-
	Chest circumference (cm)	86.549±1.600	90.444±2.998	85.992±1.601	*P*=0.342	-	-
	Chest width (cm)	21.605±0.877	21.833±1.205	20.918±0.676	*P*=0.744	-	-
	Chest depth (cm)	34.721±0.627	35.722±0.892	34.571±0.556	*P*=0.564	-	-
	Cannon circumference (cm)	8.221±0.138	8.467±0.186	8.186±0.145	*P*=0.544	-	-
FTO-5	Body height (cm)	68.081±1.013	69.065±0.593	68.076±0.952	*P*=0.591	-	-
	Back height (cm)	68.823±0.895	68.978±0.717	68.000±0.783	*P*=0.651	-	-
	Buttock height (cm)	68.468±0.806	69.728±0.732	68.606±0.879	*P*=0.449	-	-
	Body length (cm)	79.371±1.567	81.283±1.297	80.742±1.036	*P*=0.588	-	-
	Chest circumference (cm)	88.132±2.211	86.924±1.735	85.836±1.631	*P*=0.718	-	-
	Chest width (cm)	22.032±0.879	22.065±0.897	19.667±0.621	*P*=0.085	-	-
	Chest depth (cm)	34.677±0.750	35.326±0.637	34.242±0.545	*P*=0.476	-	-
	Cannon circumference (cm)	8.245±0.175	8.254±0.131	8.233±0.172	*P*=0.995	-	-
FTO-6	Body height (cm)	68.761±0.511	67.333±1.269	66.667±2.704	*P*=0.421	-	-
	Back height (cm)	68.940±0.464	67.750±1.818	65.833±2.372	*P*=0.240	-	-
	Buttock height (cm)	69.174±0.486	68.500±1.782	68.000±2.309	*P*=0.784	-	-
	Body length (cm)	80.500±0.792	82.000±3.289	79.000±2.898	*P*=0.736	-	-
	Chest circumference (cm)	87.209±1.187	87.917±3.483	80.833±0.980	*P*=0.385	-	-
	Chest width (cm)	21.457±0.563	22.417±1.069	17.333±0.422	*P*=0.125	-	-
	Chest depth (cm)	34.957±0.419	34.750±1.181	32.833±0.872	*P*=0.446	-	-
	Cannon circumference (cm)	8.237±0.099	8.450±0.238	7.967±0.440	*P*=0.578	-	-
PLIN1	Body height (cm)	-	68.389±1.382	68.511±0.498	*P*=0.924	-	-
	Back height (cm)	-	68.667±1.328	68.636±0.481	*P*=0.980	-	-
	Buttock height (cm)	-	69.444±1.322	68.957±0.492	*P*=0.698	-	-
	Body length (cm)	-	79.944±1.666	80.707±0.854	*P*=0.713	-	-
	Chest circumference (cm)	-	87.056±2.474	86.915±1.187	*P*=0.961	-	-
	Chest width (cm)	-	20.889±1.081	21.424±0.553	*P*=0.691	-	-
	Chest depth (cm)	-	34.889±0.903	34.804±0.416	*P*=0.934	-	-
	Cannon circumference (cm)	-	8.211±0.220	8.252±0.098	*P*=0.866	-	-

Notes: HS, Hu sheep; SE, standard error; II, insertion/insertion; ID, insertion/deletion; DD, deletion/deletion. [a,b]Mean values with unlike letters were significantly different, $P < 0.05$. Bonferroni multiple comparisons were performed only if there was a difference in variance analysis.

Table S3. Relationship between InDels of *FTO* and *PLIN1* and morphometric traits in DS.

Loci	Morphometric Traits	Observed Genotypes (Mean ± SE)				*Unadjusted*	*Adjusted*
		II	*ID*	*DD*	*P* values	*P* values	*P* values
FTO-2	Body height (cm)	-	67.846±1.252	67.250±5.250	*P*=0.919	-	-
	Back height (cm)	-	71.554±0.899	72.250±6.250	*P*=0.870	-	-
	Buttock height (cm)	-	67.454±0.732	72.500±3.500	*P*=0.145	-	-
	Body length (cm)	-	74.339±0.990	83.000±2.000	*P*=0.064	-	-
	Chest circumference (cm)	-	98.837±2.298	106.500±6.500	*P*=0.473	-	-
	Chest width (cm)	-	24.773±0.620	23.750±0.250	*P*=0.721	-	-
	Chest depth (cm)	-	33.959±0.705	36.500±4.500	*P*=0.447	-	-
	Cannon circumference (cm)	-	11.071±0.309	12.000±2.000	*P*=0.525	-	-
	Waist angle width (cm)	-	19.059±0.327	19.650±3.650	*P*=0.715	-	-
	Buttock width (cm)	-	20.688±0.396	21.250±2.750	*P*=0.765	-	-
	Body weight (kg)	-	54.377±0.790	53.700±4.800	*P*=0.856	-	-
FTO-3	Body height (cm)	69.850±2.626	68.073±2.051	67.091±1.801	*P*=0.751	-	-
	Back height (cm)	72.000±3.467	70.413±1.322	72.273±1.182	*P*=0.630	-	-
	Buttock height (cm)	68.867±2.756	67.527±1.220	67.477±0.933	*P*=0.815	-	-
	Body length (cm)	75.700±2.172	75.400±1.527	74.032±1.546	*P*=0.768	-	-
	Chest circumference (cm)	98.917±2.170	100.473±0.987	98.395±4.283	*P*=0.915	-	-
	Chest width (cm)	20.700±2.382[b]	26.433±0.543[a]	24.659±0.751[ab]	*P*=0.006	*P*=0.005	*P*=0.002
	Chest depth (cm)	31.783±2.301	34.753±0.913	34.241±1.036	*P*=0.399	-	-
	Cannon circumference (cm)	11.283±0.938	10.820±0.408	11.268±0.471	*P*=0.785	-	-
	Waist angle width (cm)	18.467±0.977	19.753±0.582	18.800±0.446	*P*=0.335	-	-
	Buttock width (cm)	20.917±1.165	20.600±0.575	20.736±0.592	*P*=0.967	-	-
	Body weight (kg)	53.258±2.586	52.993±1.048	55.564±1.114	*P*=0.274	-	-
FTO-4	Body height (cm)	68.441±1.647	65.250±10.250	66.986±1.620	*P*=0.774	-	-
	Back height (cm)	70.981±1.055	64.500±0.500	73.764±1.565	*P*=0.068	-	-
	Buttock height (cm)	66.715±0.853	69.750±2.750	69.271±1.402	*P*=0.221	-	-
	Body length (cm)	74.885±1.171	68.500±7.500	75.357±1.816	*P*=0.377	-	-
	Chest circumference (cm)	100.219±0.781	107.500±0.500	96.029±6.681	*P*=0.494	-	-
	Chest width (cm)	25.600±0.667	26.100±0.900	22.843±1.161	*P*=0.083	-	-
	Chest depth (cm)	33.619±0.870	35.000±3.000	34.829±1.293	*P*=0.701	-	-
	Cannon circumference (cm)	10.848±0.369	12.000±3.000	11.500±0.507	*P*=0.505	-	-
	Waist angle width (cm)	19.115±0.418	19.250±0.750	19.007±0.658	*P*=0.984	-	-
	Buttock width (cm)	20.344±0.411	19.500±2.500	21.600±0.825	*P*=0.263	-	-
	Body weight (kg)	53.320±0.906	52.150±1.850	56.636±1.441	*P*=0.111	-	-
FTO-5	Body height (cm)	66.740±2.317	67.280±2.117	69.477±1.572	*P*=0.664	-	-
	Back height (cm)	74.640±2.449	71.215±1.236	69.808±0.972	*P*=0.128	-	-
	Buttock height (cm)	69.540±1.939	67.285±1.017	66.885±1.060	*P*=0.372	-	-
	Body length (cm)	74.530±1.922	75.545±1.521	73.669±1.823	*P*=0.723	-	-
	Chest circumference (cm)	102.270±1.991	100.175±1.002	95.315±7.076	*P*=0.491	-	-
	Chest width (cm)	23.770±1.848	25.110±0.821	24.869±0.609	*P*=0.674	-	-
	Chest depth (cm)	35.580±1.794	33.750±1.076	33.423±0.835	*P*=0.492	-	-
	Cannon circumference (cm)	11.680±0.491	11.275±0.524	10.431±0.437	*P*=0.296	-	-
	Waist angle width (cm)	18.530±0.815	19.265±0.504	19.238±0.521	*P*=0.669	-	-
	Buttock width (cm)	21.170±0.942	21.040±0.608	19.862±0.498	*P*=0.358	-	-
	Body weight (kg)	57.780±1.053[a]	52.935±1.042[b]	53.873±1.598[ab]	*P*=0.039	*P*=0.037	*P*=0.012
FTO-6	Body height (cm)	67.333±1.346	67.650±3.107	76.500±2.500	*P*=0.287	-	-
	Back height (cm)	71.182±0.907	72.213±2.797	75.750±5.250	*P*=0.535	-	-
	Buttock height (cm)	67.558±0.885	67.650±1.304	70.000±3.000	*P*=0.788	-	-
	Body length (cm)	75.582±1.106	71.338±2.352	74.500±4.500	*P*=0.255	-	-

	Chest circumference (cm)	98.936±2.866	100.238±1.584	99.250±4.250	*P*=0.976	-	-
	Chest width (cm)	24.533±0.703	25.075±1.272	26.500±2.500	*P*=0.763	-	-
	Chest depth (cm)	33.873±0.747	36.438±1.042	28.000±7.000	*P*=0.051	-	-
	Cannon circumference (cm)	11.236±0.354	11.075±0.678	9.250±0.250	*P*=0.398	-	-
	Waist angle width (cm)	19.270±0.392	18.350±0.736	19.000±1.500	*P*=0.579	-	-
	Buttock width (cm)	20.939±0.457	20.338±0.747	18.500±2.000	*P*=0.388	-	-
	Body weight (kg)	54.252±0.969	54.206±0.974	56.450±3.050	*P*=0.840	-	-
PLIN1	Body height (cm)	-	68.950±1.977	67.560±1.418	*P*=0.659	-	-
	Back height (cm)	-	73.350±1.571	71.183±1.019	*P*=0.345	-	-
	Buttock height (cm)	-	67.600±1.005	67.709±0.868	*P*=0.954	-	-
	Body length (cm)	-	73.013±1.992	75.137±1.125	*P*=0.409	-	-
	Chest circumference (cm)	-	102.875±1.354	98.351±2.692	*P*=0.433	-	-
	Chest width (cm)	-	24.200±0.996	24.846±0.695	*P*=0.677	-	-
	Chest depth (cm)	-	34.425±1.108	33.997±0.819	*P*=0.814	-	-
	Cannon circumference (cm)	-	11.363±0.749	11.057±0.335	*P*=0.700	-	-
	Waist angle width (cm)	-	19.175±0.982	19.066±0.353	*P*=0.901	-	-
	Buttock width (cm)	-	21.188±1.161	20.606±0.405	*P*=0.566	-	-
	Body weight (kg)	-	57.631±1.013[a]	53.594±0.874[b]	*P*=0.040	-	-

Notes: DS, Dupor; SE, standard error; II, insertion/insertion; ID, insertion/deletion; DD, deletion/deletion. [a,b]Mean values with unlike letters were significantly different, *P* < 0.05. Bonferroni multiple comparisons were performed only if there was a difference in variance analysis.

Table S4. Relationship between InDels of *FTO* and *PLIN1* and morphometric traits in STHS.

Loci	Morphometric Traits	Observed Genotypes (Mean ± SE)				*Unadjusted*	*Adjusted*
		II	*ID*	*DD*	*P* values	*P* values	*P* values
FTO-2	Body weight (kg)	58.000±4.062	48.711±1.618	49.833±2.045	*P*=0.066	-	-
	Body height (cm)	70.375±4.616	67.790±2.208	66.111±4.108	*P*=0.790	-	-
	Body length (cm)	75.500±2.398	67.368±1.437	66.778±3.113	*P*=0.108	-	-
	Chest width (cm)	33.750±1.548[a]	26.895±1.095[b]	27.333±1.481[ab]	*P*=0.033	*P*=0.031	*P*=0.010
	Chest circumference (cm)	85.375±8.620	91.053±1.897	93.000±2.173	*P*=0.397	-	-
	Cannon circumference (cm)	11.500±0.646[a]	9.579±0.289[b]	9.222±0.465[b]	*P*=0.020	*P*=0.021	*P*=0.007
	Coccyx height (cm)	78.625±2.703	70.947±1.438	64.778±5.580	*P*=0.086	-	-
	Buttock height (cm)	72.250±3.326	65.632±1.924	65.222±2.671	*P*=0.311	-	-
	Forelimb height (cm)	29.000±3.559	25.895±2.598	24.333±2.427	*P*=0.740	-	-
	Head length (cm)	23.750±1.250[a]	19.368±0.693[b]	20.778±0.722[ab]	*P*=0.022	*P*=0.022	*P*=0.007
	Coccyx length (cm)	28.500±2.102[a]	22.632±0.714[b]	22.222±1.064[b]	*P*=0.007	*P*=0.008	*P*=0.003
	Neck length (cm)	39.625±4.879	31.526±2.953	28.778±3.337	*P*=0.324	-	-
	Forehead width (cm)	14.625±1.281	12.737±0.489	12.778±0.703	*P*=0.287	-	-
	Waist angle width (cm)	24.250±1.315	29.105±2.928	27.556±3.508	*P*=0.738	-	-
	Buttock width (cm)	18.250±0.250	20.737±0.812	20.111±1.419	*P*=0.456	-	-
	Chest depth (cm)	32.000±2.000	33.105±1.003	35.000±1.143	*P*=0.394	-	-
	Head depth (cm)	18.000±0.817	17.000±0.597	16.833±1.359	*P*=0.801	-	-
	Abdominal circumference (cm)	88.000±11.409	98.158±2.197	100.556±3.005	*P*=0.200	-	-
	Leg hip circumference (cm)	93.500±4.052	91.842±2.434	91.556±4.093	*P*=0.954	-	-
	Back height (cm)	75.125±3.332	71.474±1.634	69.444±1.668	*P*=0.364	-	-
FTO-3	Body weight (kg)	51.000±7.024	50.353±1.661	49.750±2.185	*P*=0.960	-	-
	Body height (cm)	61.333±3.180	68.912±2.193	67.417±3.560	*P*=0.503	-	-
	Body length (cm)	67.667±4.842	68.824±1.768	67.500±2.324	*P*=0.893	-	-

	Chest width (cm)	29.667±2.963^{ab}	25.765±1.006^{b}	30.417±1.417^{a}	*P*=0.030	*P*=0.032	*P*=0.011
	Chest circumference (cm)	94.000±4.583	89.265±2.547	92.417±2.190	*P*=0.563	-	-
	Cannon circumference (cm)	10.000±1.528	9.824±0.312	9.500±0.417	*P*=0.798	-	-
	Coccyx height (cm)	73.333±6.438	72.382±1.661	66.250±4.173	*P*=0.286	-	-
	Buttock height (cm)	66.333±8.090	65.588±1.667	67.417±2.612	*P*=0.848	-	-
	Forelimb height (cm)	24.000±2.517	24.471±2.042	28.250±3.547	*P*=0.571	-	-
	Head length (cm)	20.667±3.283	19.588±0.515	21.250±0.993	*P*=0.354	-	-
	Coccyx length (cm)	24.333±4.333	23.059±0.889	23.250±0.889	*P*=0.871	-	-
	Neck length (cm)	36.333±8.192	29.324±2.391	34.083±4.094	*P*=0.461	-	-
	Forehead width (cm)	13.667±1.764	13.265±0.398	12.417±0.783	*P*=0.519	-	-
	Waist angle width (cm)	24.667±1.453	27.059±2.653	30.333±3.773	*P*=0.653	-	-
	Buttock width (cm)	19.667±1.202	20.235±0.745	20.417±1.323	*P*=0.951	-	-
	Chest depth (cm)	32.333±1.856	34.177±0.924	32.833±1.370	*P*=0.613	-	-
	Head depth (cm)	17.667±1.202	17.029±0.639	17.000±1.052	*P*=0.939	-	-
	Abdominal circumference (cm)	97.333±1.202	96.059±3.357	99.750±2.947	*P*=0.722	-	-
	Leg hip circumference (cm)	92.333±6.227	92.412±2.418	91.250±3.491	*P*=0.959	-	-
	Back height (cm)	72.000±6.429	72.500±1.306	69.583±2.065	*P*=0.507	-	-
FTO-4	Body weight (kg)	49.528±1.254	-	50.346±2.650	*P*=0.783	-	-
	Body height (cm)	22.472±0.851	-	22.962±1.290	*P*=0.394	-	-
	Body length (cm)	68.722±2.591	-	65.500±2.513	*P*=0.735	-	-
	Chest width (cm)	67.667±1.444	-	68.615±2.593	*P*=0.680	-	-
	Chest circumference (cm)	27.333±1.073	-	28.077±1.491	*P*=0.682	-	-
	Cannon circumference (cm)	91.389±1.487	-	89.808±3.492	*P*=0.589	-	-
	Coccyx height (cm)	9.833±0.326	-	9.539±0.447	*P*=0.767	-	-
	Buttock height (cm)	70.389±1.482	-	69.192±4.261	*P*=0.786	-	-
	Forelimb height (cm)	66.444±2.063	-	65.615±2.126	*P*=0.109	-	-
	Head length (cm)	28.333±2.492	-	22.539±2.246	*P*=0.242	-	-
	Coccyx length (cm)	20.778±0.619	-	19.462±0.978	*P*=0.739	-	-
	Neck length (cm)	23.611±0.724	-	23.154±1.255	*P*=0.107	-	-
	Forehead width (cm)	27.889±1.752	-	34.346±3.386	*P*=0.964	-	-
	Waist angle width (cm)	13.000±0.505	-	12.962±0.676	*P*=0.114	-	-
	Buttock width (cm)	30.556±3.360	-	24.692±1.146	*P*=0.373	-	-
	Chest depth (cm)	20.722±0.999	-	19.615±0.703	*P*=0.489	-	-
	Head depth (cm)	34.056±0.822	-	33.000±1.363	*P*=0.988	-	-
	Abdominal circumference (cm)	17.056±0.769	-	17.039±0.739	*P*=0.584	-	-
	Leg hip circumference (cm)	98.167±1.942	-	95.769±4.355	*P*=0.344	-	-
	Back height (cm)	93.444±2.764	-	89.692±2.515	*P*=0.763	-	-
FTO-5	Body weight (kg)	49.111±1.940	52.500±1.897	49.900±3.172	*P*=0.543	-	-
	Body height (cm)	64.000±2.457^{b}	74.056±2.337^{a}	69.200±4.042^{ab}	*P*=0.043	*P*=0.042	*P*=0.014
	Body length (cm)	67.111±1.453	70.556±3.363	68.000±3.050	*P*=0.540	-	-
	Chest width (cm)	27.222±1.292	28.000±1.528	30.000±1.517	*P*=0.551	-	-
	Chest circumference (cm)	92.444±1.951	88.833±4.202	89.000±2.121	*P*=0.571	-	-
	Cannon circumference (cm)	9.333±0.352	10.556±0.412	9.600±0.510	*P*=0.112	-	-
	Coccyx height (cm)	68.278±3.013	74.167±2.421	69.800±3.056	*P*=0.420	-	-
	Buttock height (cm)	64.444±2.171	67.444±1.879	71.200±3.023	*P*=0.241	-	-
	Forelimb height (cm)	24.167±2.662	28.000±2.421	28.000±3.450	*P*=0.559	-	-
	Head length (cm)	20.167±0.853	21.000±0.577	19.600±1.288	*P*=0.693	-	-
	Coccyx length (cm)	23.833±0.868	22.889±1.348	21.800±1.685	*P*=0.546	-	-
	Neck length (cm)	30.222±2.283	30.833±4.188	39.000±7.836	*P*=0.344	-	-
	Forehead width (cm)	12.611±0.537	14.167±0.408	12.200±1.241	*P*=0.152	-	-
	Waist angle width (cm)	25.000±2.255	28.667±3.180	38.000±6.964	*P*=0.067	-	-
	Buttock width (cm)	19.500±0.879	21.000±1.014	21.600±1.691	*P*=0.395	-	-

	Chest depth (cm)	33.333±0.848	34.889±1.379	31.600±2.502	*P*=0.353	-	-
	Head depth (cm)	16.722±0.722	17.389±1.099	17.800±0.860	*P*=0.729	-	-
	Abdominal circumference (cm)	99.056±2.363	95.000±5.622	96.800±3.382	*P*=0.707	-	-
	Leg hip circumference (cm)	89.556±2.735	95.667±2.651	94.000±4.461	*P*=0.337	-	-
	Back height (cm)	70.722±1.709	72.833±1.379	71.000±3.728	*P*=0.740	-	-
FTO-6	Body weight (kg)	50.341±1.667	50.611±2.185	-	*P*=0.624	-	-
	Body height (cm)	68.023±2.468	67.111±2.226	-	*P*=0.917	-	-
	Body length (cm)	68.636±1.681	68.333±2.055	-	*P*=0.389	-	-
	Chest width (cm)	27.818±1.143	28.444±1.365	-	*P*=0.705	-	-
	Chest circumference (cm)	90.114±2.195	93.444±2.015	-	*P*=0.548	-	-
	Cannon circumference (cm)	9.773±0.354	9.778±0.222	-	*P*=0.495	-	-
	Coccyx height (cm)	69.159±2.603	73.222±2.146	-	*P*=0.579	-	-
	Buttock height (cm)	66.546±1.876	67.111±2.085	-	*P*=0.377	-	-
	Forelimb height (cm)	26.727±2.478	24.111±0.824	-	*P*=0.746	-	-
	Head length (cm)	20.318±0.725	20.444±0.766	-	*P*=0.909	-	-
	Coccyx length (cm)	23.091±0.761	24.000±1.453	-	*P*=0.578	-	-
	Neck length (cm)	30.477±2.029	36.111±5.496	-	*P*=0.332	-	-
	Forehead width (cm)	12.977±0.541	13.000±0.441	-	*P*=1.000	-	-
	Waist angle width (cm)	28.182±2.532	28.111±3.630	-	*P*=0.965	-	-
	Buttock width (cm)	20.000±0.830	20.667±0.972	-	*P*=0.800	-	-
	Chest depth (cm)	33.955±0.922	32.667±1.225	-	*P*=0.616	-	-
	Head depth (cm)	17.205±0.715	16.889±0.633	-	*P*=0.905	-	-
	Abdominal circumference (cm)	96.727±2.893	99.889±2.294	-	*P*=0.787	-	-
	Leg hip circumference (cm)	91.182±2.476	93.333±2.769	-	*P*=0.789	-	-
	Back height (cm)	71.386±1.470	72.333±1.833	-	*P*=0.339	-	-
PLIN1	Body weight (kg)	-	50.500±3.948	50.479±1.305	*P*=0.462	-	-
	Body height (cm)	-	70.714±3.803	67.396±2.028	*P*=0.225	-	-
	Body length (cm)	-	70.286±3.714	67.542±1.408	*P*=0.686	-	-
	Chest width (cm)	-	28.571±2.525	27.583±0.930	*P*=0.825	-	-
	Chest circumference (cm)	-	93.286±5.107	90.229±1.636	*P*=0.750	-	-
	Cannon circumference (cm)	-	10.571±0.649	9.542±0.262	*P*=0.119	-	-
	Coccyx height (cm)	-	74.857±3.348	68.938±2.302	*P*=0.436	-	-
	Buttock height (cm)	-	67.429±4.303	66.292±1.503	*P*=0.709	-	-
	Forelimb height (cm)	-	28.714±4.902	25.250±1.823	*P*=0.605	-	-
	Head length (cm)	-	21.857±1.710	20.042±0.480	*P*=0.135	-	-
	Coccyx length (cm)	-	26.143±1.682[a]	22.583±0.645[b]	*P*=0.039	-	-
	Neck length (cm)	-	34.714±5.467	30.646±2.336	*P*=0.648	-	-
	Forehead width (cm)	-	15.000±0.787[a]	12.521±0.382[b]	*P*=0.008	-	-
	Waist angle width (cm)	-	25.143±3.035	29.042±2.497	*P*=0.709	-	-
	Buttock width (cm)	-	20.000±1.175	20.417±0.771	*P*=0.795	-	-
	Chest depth (cm)	-	31.429±1.771	34.083±0.794	*P*=0.327	-	-
	Head depth (cm)	-	18.286±1.459	16.813±0.541	*P*=0.403	-	-
	Abdominal circumference (cm)	-	100.429±5.327	96.792±2.351	*P*=0.778	-	-
	Leg hip circumference (cm)	-	95.000±4.248	91.917±2.005	*P*=0.123	-	-
	Back height (cm)	-	73.143±3.377	70.938±1.223	*P*=0.703	-	-

Notes: STHS, Small Tail Han shee*P*; SE, standard error; II, insertion/insertion; ID, insertion/deletion; DD, deletion/deletion. [a,b]Mean values with unlike letters were significantly different, $P < 0.05$. Bonferroni multiple comparisons were performed only if there was a difference in variance analysis.
